# Reasons for stopping methotrexate treatment in patients with juvenile idiopathic arthritis

**DOI:** 10.1186/1546-0096-12-S1-P199

**Published:** 2014-09-17

**Authors:** Ana Carolina Da Silva, A Farias, Nailu Sinicato, R Veloso, Roberto Marini, Simone Appenzeller

**Affiliations:** 1 Medicine, State University Of Campinas, Campinas, Brazil; 2 Pediatrics, State University Of Campinas, Campinas, Brazil

## Introduction

Patients with juvenile idiopathic arthritis (JIA) commonly have been using methotrexate (MTX) to improve the clinical course of the disease, however non adherence is frequently observed.

## Objectives

To evaluate the reason for MTX interruption, if it was due therapeutic inefficacy or side effects.

## Methods

Data were collected from medical charts of JIA patients followed at the pediatric rheumatology Unit from the State University of Campinas. Relevant clinical variables was observed as age, gender, age at diagnosis, subtype of JIA, age at start of MTX, the time from diagnosis to start the treatment with MTX (in months), response to MTX (yes / no) and whether there were adverse effects (yes / no) and the reason for discontinuation of MTX.

## Results

We included 98 patients (median age 15.4 ± 7.5 years) who had a definitive diagnosis of JIA and used MTX. Seventy-six (78%) patients were females. The JIA subtype most frequently observed was polyarticular. Routes of administration of MTX observed were subcutaneous (55%) and oral (13%), but many patients throughout the treatment used both administration (32%) in order to improve adherence to treatment. After a mean follow-up time of 5 years, 51 (52%) of these patients discontinued treatment and 47 (48%) of them were still on treatment, but only 35 (74%) were adherent. Sixty-eight (69%) JIA patients reported the presence of sporadic episodes of adverse reactions during treatment. Episodes of adverse reactions related to MTX was mainly gastric intolerance (abdominal discomfort, nausea and vomiting). There were three cases of elevated transaminase levels and one case of neutropenia. The reasons for JIA patients discontinue treatment with MTX was presence of side effects (35%), interruption on their own decision (27%), treatment abandonment (12%) and other reasons such as pregnancy, chickenpox and lack of medication in the public hospitals (12%), improvement of the disease (8%), and inefficacy (6%). All patients who discontinued MTX on their own decision had additionally the presence of side effects.

## Conclusion

Although MTX has a great therapeutic index, the side effects are still seen as a major form of abandonment of this pharmacological treatment. Therefore a careful history is essential to identify side effects and adequate treatment to increase adherence.

## Disclosure of interest

A. C. Da Silva: None declared, A. Farias: None declared, N. Sinicato: None declared, R. Veloso: None declared, R. Marini: None declared, S. Appenzeller Grant / Research Support from: FAPESP (2008/02917-0, 2011/03788-2, 2013/09480-5) CNPQ (300447/2009-4 and 471343/2011-0; 302205/2012-8; 473328/2013-5).

